# Green H_2_ Production by Water Electrolysis Using Cation Exchange Membrane: Insights on Activation and Ohmic Polarization Phenomena

**DOI:** 10.3390/membranes12010015

**Published:** 2021-12-23

**Authors:** Elisa Esposito, Angelo Minotti, Enrica Fontananova, Mariagiulia Longo, Johannnes Carolus Jansen, Alberto Figoli

**Affiliations:** 1 Institute on Membrane Technology, CNR-ITM, Via P. Bucci 17/C, 87036 Rende, Italy; e.esposito@itm.cnr.it (E.E.); m.longo@itm.cnr.it (M.L.); johannescarolus.jansen@cnr.it (J.C.J.); a.figoli@itm.cnr.it (A.F.); 2 MIPRONS srl, Via Lauri 32, 00037 Segni, Italy

**Keywords:** electrolysis, green hydrogen, O_2_ production, proton exchange membrane, renewable energy, ohmic and activation resistances

## Abstract

Low-temperature electrolysis by using polymer electrolyte membranes (PEM) can play an important role in hydrogen energy transition. This work presents a study on the performance of a proton exchange membrane in the water electrolysis process at room temperature and atmospheric pressure. In the perspective of applications that need a device with small volume and low weight, a miniaturized electrolysis cell with a 36 cm^2^ active area of PEM over a total surface area of 76 cm^2^ of the device was used. H_2_ and O_2_ production rates, electrical power, energy efficiency, Faradaic efficiency and polarization curves were determined for all experiments. The effects of different parameters such as clamping pressure and materials of the electrodes on polarization phenomena were studied. The PEM used was a catalyst-coated membrane (Ir-Pt-Nafion™ 117 CCM). The maximum H_2_ production was about 0.02 g min^−1^ with a current density of 1.1 A cm^−2^ and a current power about 280 W. Clamping pressure and the type of electrode materials strongly influence the activation and ohmic polarization phenomena. High clamping pressure and electrodes in titanium compared to carbon electrodes improve the cell performance, and this results in lower ohmic and activation resistances.

## 1. Introduction

Hydrogen produced without CO_2_ emissions can play an important role in the next years for reaching the target of decarbonization and climate neutrality. In March 2020, the European Commission proposed the Clean Hydrogen Alliance (CHA) as a strategy for developing a network between research, private companies and public institutions to promote hydrogen technology [1,2,3]. Over the next 30 years, hydrogen can drive the green revolution thanks to its endless potential applications in the industrial energy and transport sectors. The “hydrogen economy” can be the solution to environmental problems and a strategy for zero greenhouse gas (GHG) emissions by 2050 [4].

Hydrogen can be produced by a variety of processes, with or without associated greenhouse gas emissions, depending on the technology and energy source used [5]. “Grey hydrogen” is produced by fossil fuels (mostly natural gas and coal), causing emission of carbon dioxide, while “blue hydrogen” is produced from natural gas or steam reforming processes, combined with carbon capture and storage procedures (CCS) [6]. Today, 73.9 million tons of hydrogen are produced in the world, of which 96% comes from grey and blue hydrogen, while only 4% of the produced hydrogen is green. From a climatic and environmentally friendly point of view, “green” hydrogen, generated by electrolysis of water, using electricity from renewable energy sources such as solar photovoltaic, wind and hydropower, is the most interesting, with the production of only oxygen as a byproduct. The additional advantage of green hydrogen is that it can store the excess of renewable energy over days, allowing consumers to use the surplus of renewable energy converted, even when there is no wind or sun [7]. In fact, hydrogen is recognized as an energy carrier and it can deliver or store a large amount of energy. Moreover, the combination of Proton Exchange Membrane (PEM) fuel cells and PEM electrolyzers provides a back-up system for renewable energy sources, avoiding intermittency: electrolyzers convert the excess of energy from renewable energy sources into hydrogen, and PEM fuel cells use this hydrogen to convert it back into electricity when it is needed [8]. The current cost of grey and blue hydrogen is between 1.20 USD and 2.40 USD/kg depending on the cost of CCS [9]. On the other hand, the cost of green hydrogen is still high, about 6.00 USD (5.09 EUR) per kilogram of H_2_ produced [3], more than half of the cost of traditional electrolyzers is given by the stack investment [10]. However, with decreasing cost of electrolyzer elements and improving efficiency of PEM membranes (PEM), electrolyzers can reduce the price of green hydrogen down to less than 2.00 USD/kg by 2030, becoming competitive with grey/blue hydrogen [11].

The first proton exchange membrane (PEM) electrolyzer was used in space for the Gemini mission (NASA) in 1960, and still today, electrolysis is attractive in the aerospace sector because hydrogen is one of the main fuel for spacecrafts, and oxygen is necessary for all manned missions [12,13,14]. Since then, the use of hydrogen in the aviation and aerospace industry has received big interest. In 1988, the Soviet Union launched the Tupolev Tu-155 project, which was the first experimental aircraft in the world operating with hydrogen [15]. Consequently, a number of different projects based on the use of hydrogen as a propellant were proposed, such as CRYOPLANE (Europe- 2000), Hy-Shot (Australia- 2001), NASA X-43 (USA- 2004) and Phantom Eye (USA- 2013) [5]. The use of hydrogen for aviation and space exploration has the potential to significantly reduce aviation’s climate impact. At the same time, the development of this technology for space exploration, where reduced size and weight are extremely important, can be the incentive for improving the efficiency of the electrolyzer and reducing its cost–efficiency ratio compared to that of grey and blue hydrogen. 

Different technologies are available for water electrolysis, and each is in a different development stage. Alkaline electrolyzers represent the state of the art, and proton exchange membrane (PEM) technologies are in a developmental stage, while solid oxide electrolyzers are still in the research and development phase [6,16]. PEM electrolyzers may provide a range of advantages compared to the other electrolysis technologies. It can operate at much higher current densities, reducing the operational cost and the overall cost of electrolysis. PEM electrolyzers work under a wide range of power input, responding quickly to the power input. Finally, a solid membrane electrolyte allows a compact and mechanically resistant system design, suitable for operation at high pressures [4]. The maximum amount of hydrogen produced is directly correlated to the current, but the power needed is the product of the current and the voltage over the membrane cell. Therefore, in the evaluation of PEM electrolysis performance, the polarization curves provide important information of the energy efficiency on the system. In 1973, Russel et al. presented the first study on the PEM performance in the electrolysis process [17]. They reported the first voltage-current profile (polarization curve) at 48.8 °C. Since then, many other works were reported, usually operating above room temperature [18] because electrolysis at elevated temperatures has kinetic and thermodynamic advantages in the water splitting reaction [19]. In most studies about PEM performance, an excess of water is supplied to the system and recirculated to remove any waste heat [17]. Moreover, the water temperature is maintained at 80 °C for facilitating the water splitting reaction [20,21,22], and many studies are carried out on large dimensions and heavy electrolyzers [23,24]. However, low temperature PEM electrolysis is one of the most promising candidates for producing renewable hydrogen with a lower investment in energy consumption [25]. In 2012, N. Mamaca et al. evaluated the electrolysis performance of a 50 cm^2^ Nafion™ 115 CCM at room temperature and atmospheric pressure by using a low current (1–5 A) [26]. They were able to feed 5 A, obtaining a cell voltage of 2.44 V. Recently, Selamet et al. reported a study of five-cell stack and ten-cell stack electrolyzers at room temperature [23]. The cell stacks showed a voltage of about 10 and 19 V, respectively, for a current density of 1 A cm^−2^ at 20 °C. The scope of all researchers in the PEM electrolysis process is to reduce the voltage-current profile in order to obtain a high energy efficiency of the electrolyzer [27]. In this work, we investigated the performance of a small and lightweight electrolyzer in view of a potential use in space application, in which small volume and low weight are required. For size and practical reasons, the system had no water recirculation and used a high electrical current to reach high hydrogen production. A catalyst-coated membrane (CCM) was characterized simultaneously under the following conditions: without a water recirculation system and without providing external heat in miniaturized electrolyzer, by using a wide current range (1–40 A). We characterized the system, in terms of H_2_ production, energy efficiency and Faradaic efficiency. The final aim was to study polarization phenomena that can occur during the water electrolysis process and to understand the key factors that can mitigate these phenomena.

## 2. PEM Electrolysis Background

### 2.1. Principle of PEM in Water Electrolysis

**Figure 1:**
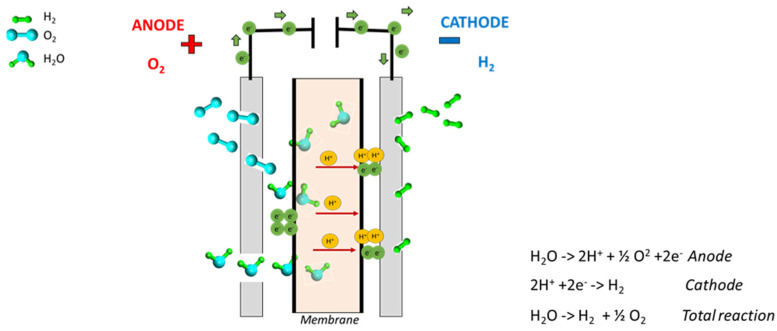
Working principle of a PEM electrolyzer.

In the PEM water electrolysis process, water is electrochemically split into hydrogen and oxygen at their respective electrodes: hydrogen at the cathode and oxygen at the anode side. The core of the process is the membrane, which ensures the efficient transport of protons, working as an electrically insulating barrier for the gases produced (and for electrons). The water is fed into the anode side where it is spilt into oxygen (O_2_), protons (H^+^) and electrons (e^−^) for the oxygen evolution reaction (OER). These protons move via proton exchange membrane to the cathode side. The transport of protons is closely related to the water transport through the membrane. The most accepted modes of membrane protons’ transport is the Grotthuss mechanism, in which hydronium (H_3_O^+^) is formed by a hydrated sulfonate group, contained in the polymer structure, and subsequently jumps to the next sulfonate group combined with a vehicular mechanism in which protons move through the membrane together with the water hydration molecules, until being released at the cathode side [28]. The electrons leave the anode through the external power circuit, which provides the driving force (cell voltage) for the hydrogen evolution reaction (HER). At the cathode side, the protons and electrons recombine to produce hydrogen, following the mechanism shown in Figure 1. (3) In a PEM electrolyzer, key components are the electrodes working as current collectors and a gas diffusion layer. Water diffuses via these electrodes at the anode side while gases produced at the catalyst coated layers outgone the cell through them. Moreover, they must be providing an electron conduction path. The corrosive environment due to the acidic environment, high over potential and presence of oxygen (at anode side) create specific issues in electrode material selection, and current collectors together with separator plates are responsible for about 48% of the cell capital cost [4]. Typically, in PEM water electrolysis, porous titanium plates are used due to their good electrical conductivity, elevated mechanical stability and high corrosion resistance under acidic conditions [29,30]. In this work, we compared titanium electrode with carbon electrodes at the cathode side.

Moreover, PEMs for the electrolysis process must possess the following desirable properties: high proton conductivity to reduce ionic transport resistance, electronic insulating properties, adequate mechanical and chemical stability under long-term operating conditions, low crossover of oxygen and hydrogen, and production costs compatible with the intended application. The membranes usually used in the water electrolysis are membranes based on perfluorosulfonic acid polymers (PFSA) because they guarantee high proton conductivity conjugated with elevated chemical resistance [31].

PFSA with different chemistry (long and short and short side chain) and thickness (from tens to hundreds of micrometers) are commercially available today and show strong structure/property relationships [32,33,34]. One of the main limitations of PFSA membranes is their elevated cost due to the complex fluorine chemistry involved in their fabrication. However, despite the numerous lab-scale investigations on nonfluorinated membranes for fuel cells and membrane electrolyzers, they still remain benchmark materials for these applications due to their unique transport properties and stability. Thinner PFSA could reduce ionic transport resistance and, consequently, energetic efficiency of the electrolyzer. An additional advantage in the use of thinner membranes is their lower cost. However, they could also increase reactant crossover and durability issues (pinhole defects). In this work, a Nafion 117 membrane with a thickness of 180 µm was selected as the cation exchange membrane for water electrolysis because it offered a good compromise between ionic conduction, barrier properties and chemical resistance over a long time.

Water splitting is an endothermic reaction and can be schematized as follows [4]:

H_2_O (l) + ΔH → H_2_ (gas) + ½ O_2_ (1)

where, ΔH is the enthalpy of the reaction, which must be provided to the water for dissociating into hydrogen and oxygen. The enthalpy of the reaction can be calculated as:

ΔH = ΔG + *T* ΔS (2)

where ΔG is Gibbs free energy, *T* is temperature (K) and ΔS is entropy of the reaction. Gibbs free energy is the minimum energy required to decompose the water, and is reversible. The minimum voltage needed to provide the energy required for water splitting is about 1.48 V [35].

In water electrolysis, Faradaic efficiency is a quantitative measure used to express how many electrons are transported via the external circuit to the surface of the electrode, in relation to how many are needed to conduct the electrochemical reaction. Therefore, Faradaic efficiency can be defined as the ratio between experimental volume of gas produced (hydrogen or oxygen) and the theoretical calculated volume of gas, as follows

(3) $$\eta_{Faraday}\left(\%\right)=\frac{V_{H_{2}\;\left(produced\right)}\;}{V_{H_{2}\;\left(theoretical\right)}}\times 100$$

where *V_H_*_2_ theoretical is calculated from Faraday’s second law:

(4) $$V_{H_{2}\;\left(theoretical\right)}=\frac{V_{M}\cdot I\cdot t}{2F}$$

where *V_M_* is molar volume of the gas (L mol^−1^), *T* is temperature expressed in Kelvin, P is pressure (atm), *t* is time (s), *I* is applied current (A) and *F* is the Faraday constant (96.485·10^3^ s A mol^−1^).

The energy efficiency of the system is calculated from the ratio between the energy that can be produced by the combustion of hydrogen formed and energy consumed in the electrolytic process as reported in the following equation

(5) $$\eta_{E}\left(\%\right)=\frac{Energy\;produced}{Energy\;used}\;\times 100$$

The energy produced is calculated as the product of the quantity of hydrogen formed per unit of time and its higher heating value (HHV_H2_ = 41.8 MJoule kg^−1^). The energy used corresponds to the electrical power *P*.

(6) P=I×V

This is the energy necessary for transferring the electrons in an electric circuit thanks to the driving force generated by the voltage difference. The cell voltage, *V*, is the driving force of the reaction and yields the resistance of the cell-membrane system via Ohm’s first law:

(7) $$R=\frac{V}{I}$$

where *R* is the resistance (Ohm).

### 2.2. Polarization Curve for PEM Electrolysis

**Figure 2:**
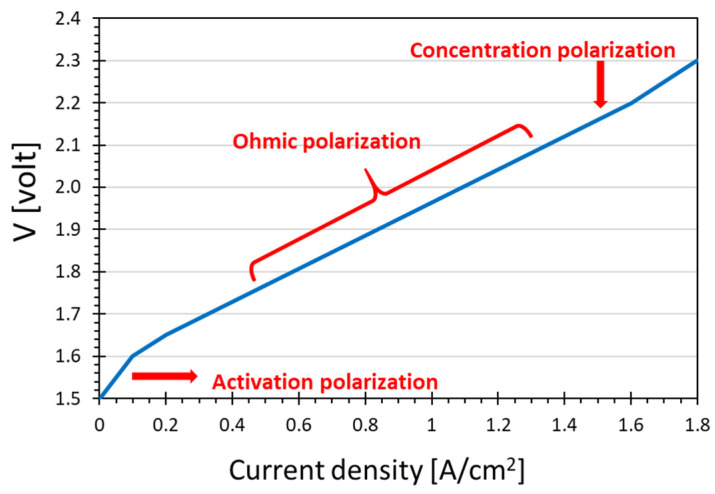
Typical polarization curve for a PEM electrolyzer.

In PEM electrolysis processes, the hydrogen production rate is directly related to current drawn by the electrolyzer. However, polarization phenomena make that the operating voltage is not constant but increases with the current, and the power consumption of the cell does not increase linearly but increases more and more at high current densities. The increase of the voltage as a function of the current density determines the so-called polarization curve, shown in Figure 2. Three distinct regions exist, each of them related to one of the three different polarization types.

The initial increase step is attributed to the barrier for the electron transfer reactions occurring at the electrodes. This is referred as the “activation polarization region” [36]. As the load resistance is decreased further (i.e., at increased current density), there is a range where the voltage increases almost linearly with the current. This is referred to as the “ohmic polarization region”, where the current is limited by the internal resistance to ion flow [36]. With further increasing current, it reaches a limiting value where the mass transfer of reactants to the electrode/electrolyte interface limits the reaction. This is known as “concentration polarization region” [37]. Cells that exhibit nonlinear behaviour at higher currents exhibit polarization, and the degree of polarization is given by an overvoltage, or overpotential, which represents the difference between the theoretical and effective potential required.

## 3. Materials and Methods

### 3.1. Proton Exchange Membrane

**Figure 3:**
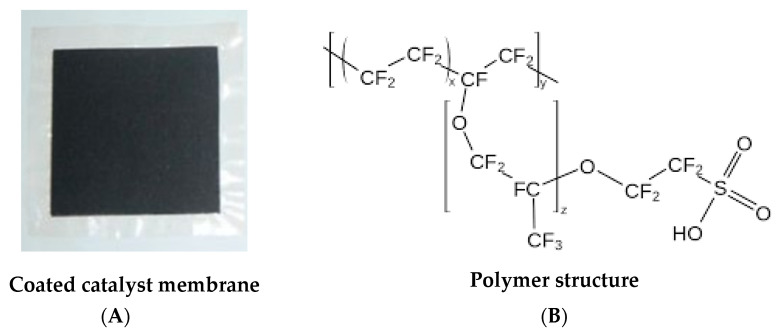
(**A**) Image of Ir-Pt-CCM of Nafion™ 117; (**B**) chemical structure of Nafion™ 117.

The catalyst-coated membrane (CCM) used for electrolysis test was a commercial Nafion™ 117-based proton exchange membrane purchased from *Quintech* (Figure 3A,B). The membrane contained 1 mg cm^−2^ of platinum (Pt) on the cathode side and 2 mg cm^−2^ of iridium (Ir) with carbon on the anode side and is abbreviated as Ir-Pt-CCM of Nafion™ 117.

### 3.2. Electrodes

Two different electrodes for the cathode side were used. The titanium electrode (Titanium-E) was characterized by an Ohmic resistance of 0.2 Ohm, and the carbon electrode (Carbon-E) had an Ohmic resistance of 1.4 Ohm (Table 1). Both resistances were an average of the Ohmic resistances calculated by means of an Ohmmeter device in ten different points of the electrode surface. Titanium-E and Carbon-E were both purchased from *Quintech*.

### 3.3. Electrolysis Setup

**Figure 4:**
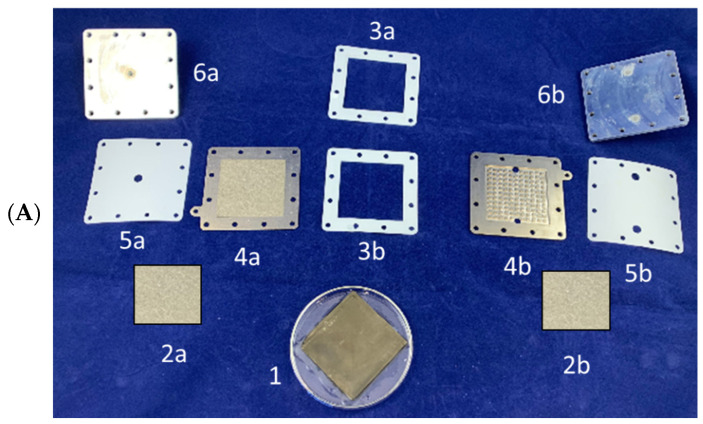

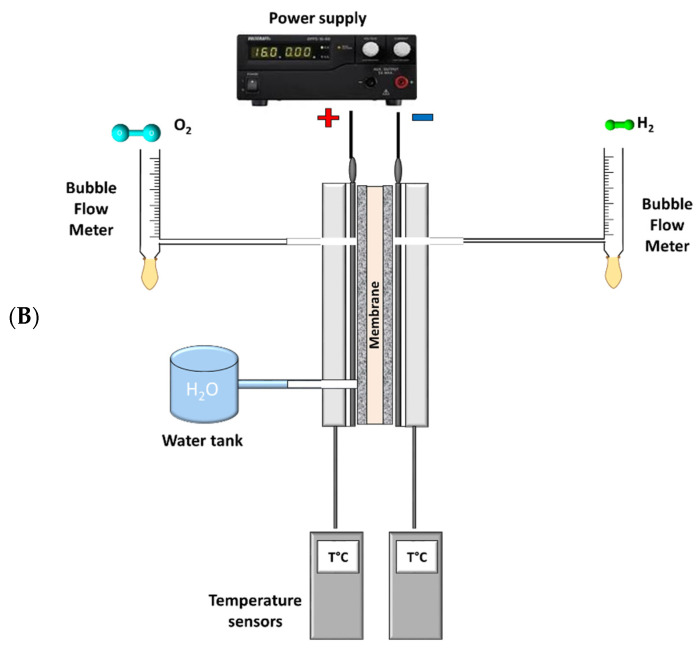
(**A**) Electrolyzer cell elements: CCM (1), titanium porous electrodes (2a, 2b), teflon seals (3a, 3b, 5a, 5b), cathodic (4a) and anodic (4b) current collectors, end plates (6a, 6b). (**B**) Schematic view of the experimental setup for the electrolysis tests.

The electrolysis cell was provided by DeltaE S.r.l. and had small dimensions with a total size of 87 × 87 × 15 mm and a weight of ca. 200 g. Figure 4 shows the components of the electrolyzer with a schematic representation of the electrolyzer setup. The effective area of the Ir-Pt-Nafion™ 117 CCM was 36 cm^2^ and was in contact with two porous titanium electrodes that worked as anode and cathode, respectively. The electrodes were in direct contact with the current collectors. A Voltcraft DPPS-16-60 power source was used to provide the electrical current. Teflon seals and terminal aluminium plates with 12 bolts ensured a homogenous compression of the cell. The anode side presented an inlet and outlet connections for feeding water and collecting O_2_, respectively. The supply of water was provided when needed by means of a prefilled syringe. The cathode side had a single outlet to collect the produced H_2_. The produced gases diffused away from the membrane through the two porous titanium electrodes. The desired current was set, and the resulting voltage was recorded during each experiment using the SLAB software program. The temperature was externally measured on the surfaces of the the anode and cathode side with thermocouples. The gas flows were measured manually with bubble flow meters and were converted into STP units, taking into account the atmospheric temperature and pressure during each measurement.

### 3.4. Chemical and Morphological PEM Analysis

Chemical and morphological analysis of the membrane was performed by scanning electron microscopy (Phenom Pro X desktop SEM, Phenom-World). Elemental analysis was performed with the built-in energy dispersive X-ray spectroscopy detector (EDX). The samples for analysis of the cross-section were freeze-fractured in liquid nitrogen.

## 4. Results and Discussion

### 4.1. Chemical and Morphological Characterization of PEM

**Figure 5:**
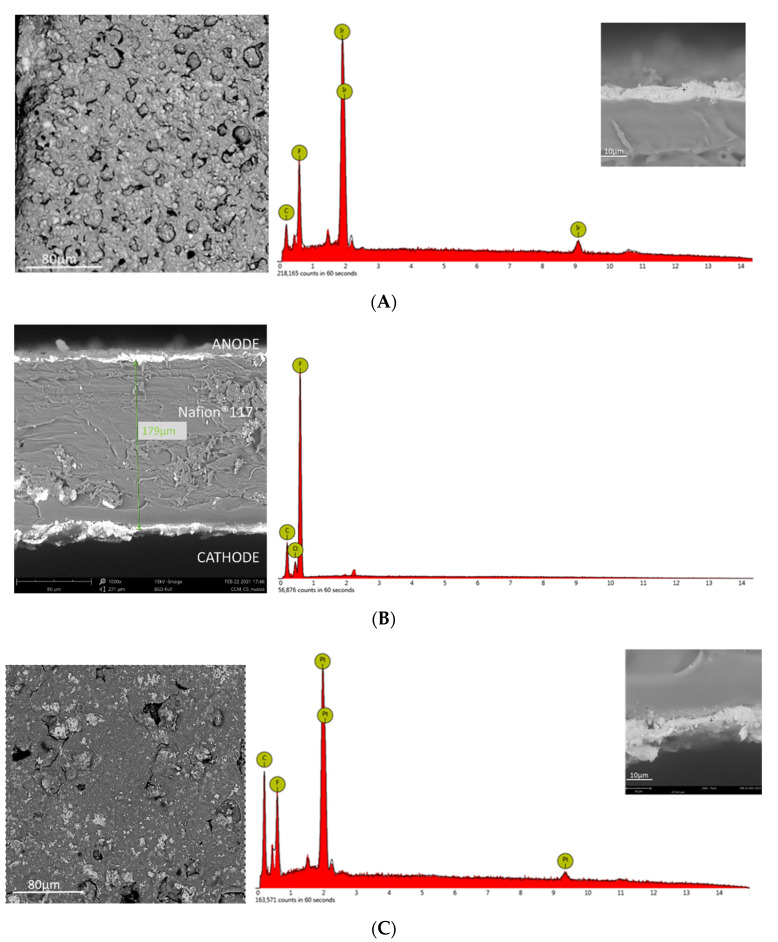
SEM and EDX analysis of the surface at a magnification of 500× and an accelerating voltage of 15 kV for the anode side (**A**) and cathode side (**B**), and cross-section (**C**) of CCM at magnification of 1000× and an accelerating voltage of 15 kV for the Ir-Pt-Nafion™ 117 CCM.

Figure 5 shows the surface morphology of the anode catalyst layer (Ir catalyst mixed with Nafion™ ionomer) and the cathode catalyst layer (Pt/C catalyst mixed with Nafion™ ionomer). The surface of the catalyst layer showed a cauliflower morphology, typical for this kind of membranes in which the surface is coated with a metal catalyst [38] (Figure 5A,C). The cross-section of the membrane shows a thickness of about 179 μm for the Nafion™ 117 polymeric layer, and a few microns for the iridium and platinum catalyst layers (Figure 5B). The EDX analysis showed the typical peaks of iridium on the anode side and platinum on the cathode side (Figure 5A,C, respectively).

### 4.2. Electrolysis Performance

**Figure 6:**
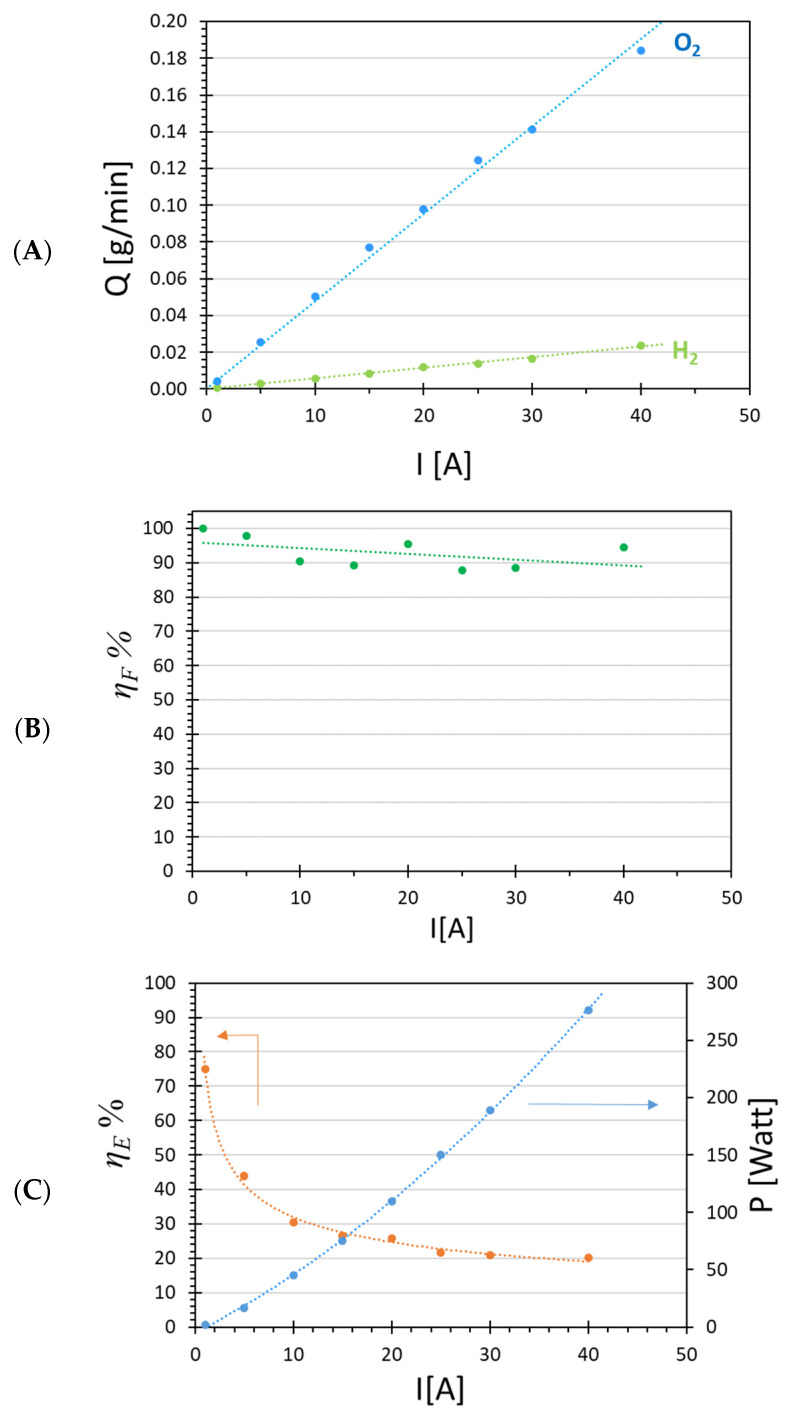
Flow rate of H_2_ and O_2_ produced (**A**), Faradaic efficiency (**B**), energy efficiency and electrical power (**C**) as function of electrical current applied for the Ir-Pt-Nafion™ 117 CCM.

Figure 6A shows the H_2_ and O_2_ production rate as a function of the electrical current for the Ir-Pt-Nafion™ 117 CCM. Increasing the current from 1 to 40 A yielded a proportional increase in H_2_ and O_2_ production, up to the maximum value of 0.024 g min^−1^ and 0.18 g min^−1^, respectively. Over the entire range, these gas productions as a function of the current were close to the theoretical amount of gas produced according to (4), and the Faradaic efficiency varied in the range between 88% and 98% (Figure 6B).

**Figure 7:**
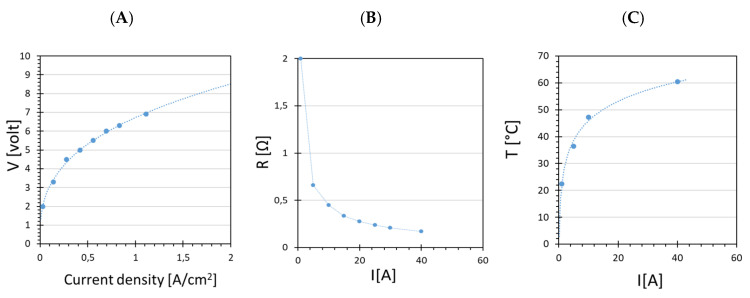
(**A**) Polarization curve for the Ir-Pt-Nafion™ 117 CCM electrolyzer test; (**B**) resistance of the system as function of the current; (**C**) temperature increase as function of the current.

Slight fluctuations in the Faradaic efficiency were probably related to the change in temperature that occured during increased electrical current run (Figure 7C).

While increasing the electrical current from 1 to 40 A, cell temperature increased from 20 to 60 °C (Figure 7C). The increase in temperature was due to the system absorbing electrical power and converting it into heat. During operation at high current density and well above the thermoneutral potential, all the heat is provided by the internal production of thermal energy as a consequence of the exothermic process [19]. For this reason, greater electrical power was necessary for conducting the reaction, which increased rapidly with increasing electrical current, from 5 W at 1 A to 280 W at 40 A (Figure 6C). Consequently, the energy efficiency calculated by (5) decreased from an initial value of 75% to 25% at the highest current (Figure 6C).

The decreased energy efficiency was a direct consequence of the increased cell voltage, shown in the polarization curve (Figure 7A), which caused a nonlinear increase in the required power, while the hydrogen production increased linearly. The Ohmic resistance decreased as a function of the current density. This was due to the increased temperature, which decreased the ohmic membrane resistance and increased the kinetic diffusion of protons trough the membrane (dragging effect) [39]. In our case, we could assume that there was no significant concentration polarization because we did not observe significant loss in hydrogen production, and almost all reactants (electrons and protons) were converted into products (H_2_ and O_2_). However, an activation polarization type and an ohmic polarization phenomena took place, as in Figure 7A. Ohmic polarization occurs when the reactant species do not reach the surface of the electrode quick enough or the species produced do not move away from it fast enough to maintain the desired current. This behaviour is usually due to the presence of a gap in the transport of electrons from the current collectors to the electrodes on the membrane surface, and the resulting higher voltage generates extra heat, according to (6). This gap in electron transport is due to poor interface contacts between current collectors and electrodes on the membrane surface [35]. Moreover, the overpotential observed in the activation phase highlighted the need for 2 V, instead of the theoretical value for activation of electrolysis reaction, equal to 1.48 V. This difference of about 0.55 V could be due to the resistivity of the gas diffusion layers, and resistivity of the membrane, each typically contributing more than 0.3 V over the theoretical voltage [40,41].

### 4.3. Effect of Clamping Pressure

Because of the high voltages observed, different clamping pressures of 11, 23 and 34 bar were applied to the electrolyzer cell in order to minimize the interface resistances and to improve the electrolysis performance. The clamping pressure (*Pc*) can be calculated from clamping force (*Fc*) applied on the cell, divided by the area of the cell.

(8) $$P_{c}=F_{c}/A_{c}$$

The clamping force was calculated from the sum of the force exerted by each bolt, by the following equation:

(9) $$F_{c}=N\times \tau /f\times D$$

where *N* is the number of bolts symmetrically distributed over the area of the cell, *τ* is the torque applied to each bolt (*N*⋅m) by a torque wrench, *f* is the friction coefficient (0.2 for steel bolts), and D is the nominal bolt diameter (m) [28].

**Figure 8:**
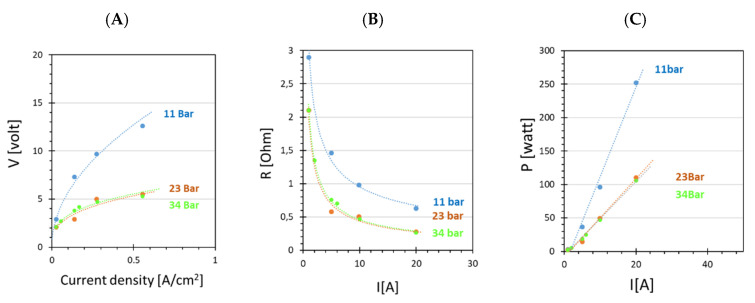
Polarization curve (**A**); resistance of electrolyzer (**B**) and electric power (**C**) as a function of current for the Ir-Pt-Nafion™ 117 CCM at three different clamping pressures.

The cell voltage necessary for activating the electrolytic reaction decreased from 3 to 2 V by increasing the clamping pressure from 11 to 34 bar. Moreover, kinetic polarization was drastically reduced at a higher clamping pressure, especially when a high current was applied (Figure 8A). According to Selamet’s work about the effects of bolt torque [42], higher clamping pressure can reduce the resistance to the transport of ionic species, thanks to the tightening of the air gap between the cell components and to a better contact between the membrane and the electrodes. The lower resistance at higher clamping pressure (Figure 8B) reduced the electrical power P needed to generate H_2_, especially at a high current (Figure 8C).

**Figure 9:**
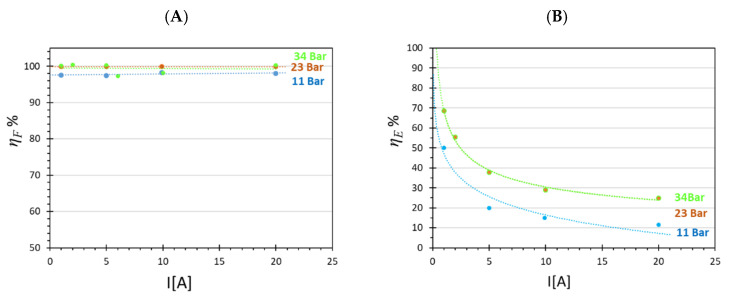
(**A**) Faradaic efficiency (**B**) and energety efficiency as a function of the current for the Ir-Pt-CCM at three different clamping pressures.

The increase in clamping pressure from 11 to 23 bar caused also a very small increase in Faradaic efficiency and a larger one in energy efficiency (Figure 9). The latter became similar at 34 and 23 bar as expected, energy efficiency being the direct consequence of electrical power consumed. The high Faradaic efficiency in all experiments confirmed that H_2_ crossover was negligible.

### 4.4. Effect of Electrode Material

**Figure 10:**
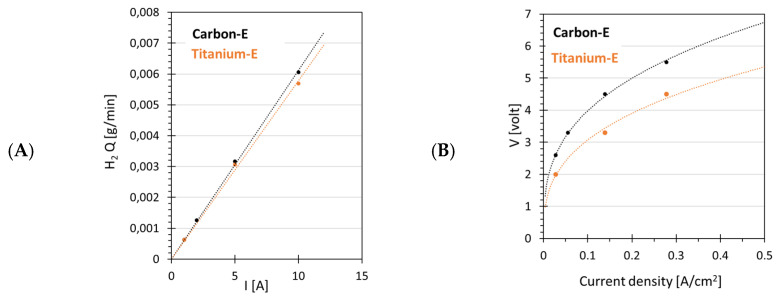

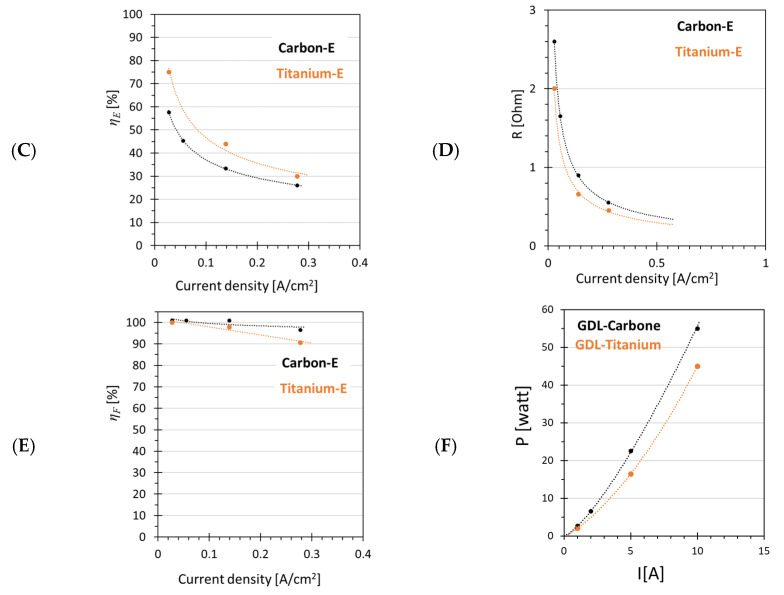
Effect of the cathode electrode material on the H_2_ production rate (**A**), energy efficiency (**C**), Faradaic efficiency (**E**), polarization curve (**B**), resistance (**D**) and electrical power (**F**) as a function of electrical current (1 A, 2 A and 5 A and 10 A).

The effect of the electrode materials was investigated by replacing the titanium electrode (Titanium-E) on the cathode side with a carbon cloth electrode (Carbon-E). H_2_ production as a function of the current was similar for both electrodes (Figure 10A), while the type of electrodes significantly affected the polarization curve (Figure 10B). Voltages were higher for Carbon-E compared to those for Titanium-E for all current values. This suggests a higher system resistance when the Carbon-E type was used (Figure 10B). Indeed, the Carbon-E had a resistance of about 1.4 Ohm, significantly higher compared to the 0.2 Ohm of the Titanium-E type electrode. The higher electrical power needed for Carbon-E with respect to the Titanium-E, the lower the energy efficiency for Carbon-E (Figure 10C,F). With Titanium-E, the energy efficiency decreased from an initial value of 80% to 30%, while for Carbon-E, the initial energy efficiency was already slightly lower (about 57%) and further decreased to 25% at a current density of 0.28 A cm^−2^. On the other hand, the Faradaic efficiency was slightly higher for Carbon-E, about 100%, and was almost constant with increasing current density. This slightly higher energy efficiency for Carbon-E could be ascribed to the hydrophobicity of Carbon-GDL, which may not interact with the amount of water available on the membrane surface, preventing phenomena such as oxidation of gas diffusion layer or cathode flooding and improving Faradaic efficiency [41,43].

## 5. Conclusions

The performance of a lightweight electrolyzer was studied in view of potential applications where the size and weight of the system can be critical. The work demonstrated that a 36 cm^2^ active area of Ir-Pt-Nafion™ 117 CCM yielded 0.024 g min^−1^ of hydrogen at the highest current density of 1.1 A cm^−2^ with an energy efficiency of 20%. The maximum energy efficiency of 75% was obtained at the lowest current density of 0.03 A cm^−2^. The system without water recirculation generated heat which favored the reaction, reducing the ohmic resistance. Activation polarization was observed with an overpotential of 2 V and an ohmic polarization with overpotential that increased with the increase of electrical current. The present study on PEM water electrolysis performance showed that the following conditions are recommended in order to reduce the activation polarization, the ohmic overpotential and, as a consequence, to increase the energy efficiency.

A sufficiently high clamping pressure is necessary in order to optimize the interface contact between the membrane, electrodes and current collector, to avoid a gap between the electrolyzer components for the transport of ionic species and to yield high energy efficiency. The increase of clamping pressure from 15 to 23 bar allowed reaching an increment in the energy efficiency of about 20% at the lowest current density of 0.03 Acm^−2^ and at the highest current density of 0.5 Acm^−2^. Poor contact between the current collector, membrane and electrodes causes ohmic polarization phenomena, decreasing PEM electrolysis’ performance in terms of energy efficiency. This effect is more evident at high electrical currents because of the higher number of electrons and protons. At the same time, good contact between the current collector, membrane and electrodes decreased the voltage necessary to activate the water splitting reaction mitigating the activation polarization.

Even the electrode materials influenced both the activation polarization and ohmic polarization. Carbon-E had resistance higher compared to that of Titanium-E and showed higher voltage in the region of activation polarization and ohmic polarization. Thus, electrodes need to have low resistance to guarantee high efficiency of the electrolysis process. The miniaturized electrolyzer for operation without water recycling and providing external heat needs to work at a low current density to yield high energy efficiency. On the other hand, a high membrane area is necessary to obtain elevated hydrogen production at a low current density. For this reason, a new membrane electrolyzer design is necessary in order to improve space packing of membranes and provide a prospective on low-temperature water electrolysis.

## Figures and Tables

**Table 1:** Types of electrodes used for the cathode side.

Code Name	Picture	Ohmic Resistance, (Ohm)	Thickness, (µm)
Titanium-E	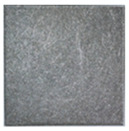	0.2	250
Carbon-E	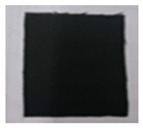	1.4	250

## Data Availability

Not applicable.
